# Validation of Body Surface Area Equations for Estimating Fat-Free Mass by Dual X-Ray Absorptiometry in a Regional Chilean Sample Aged 4 to 85 Years

**DOI:** 10.3390/diagnostics15232982

**Published:** 2025-11-24

**Authors:** Marco Cossio-Bolaños, Rubén Vidal Espinoza, Jose Sulla-Torres, Camilo Urra-Albornoz, Lucila Sanchez-Macedo, Miguel de Arruda, Fernando Alvear-Vasquez, Evandro Lazari, Rossana Gomez-Campos

**Affiliations:** 1 Departamento de Ciencias de la Actividad Física, Universidad Católica del Maule, Talca 3460000, Chile; 2 Facultad de Educación, Universidad Católica Silva Henriquez, Santiago 8330000, Chile; 3 Escuela Profesional de Ingenieria de Sistemas, Facultad de Ciencias e Ingenierías Físicas y Formales, Universidad Católica de Santa María, Arequipa 04001, Peru; 4 Departamento de Ciencias de la Educación, Facultad de Educación y Humanidades, Universidad del Bío Bío, Chillán 3780000, Chile; 5 Escuela Profesional de Educación Física, Facultad de Ciencias de la Educación, Universidad Nacional del Altiplano de Puno, Puno 21001, Peru; 6 School of Physical Education, State University of Campinas, Campinas 13083-851, Sao Paulo, Brazil; 7 Escuela de Ciencias del Deporte y Actividad Física, Facultad de Salud, Universidad Santo Tomás, Talca 3460000, Chile

**Keywords:** lean body mass, body surface area, DXA, Chile

## Abstract

**Background/Objectives:** Body surface area (BSA) is an important metric that represents human dimensionality and could provide a more accurate representation of body composition. The objectives were (a) to verify the validity of a set of equations based on BSA to estimate lean body mass (LBM), using dual X-ray absorptiometry (DXA) as a reference method and (b) to propose reference values of BSA by anthropometry and LBM by DXA in a regional sample of Chile aged 4 to 85 years. **Methods:** A descriptive cross-sectional study was performed. The sample size was 5493 participants. Weight and height were measured. BSA was calculated using seven equations. LBM was assessed by DXA. **Results:** Only three BSA equations (Dubois–Dubois, 1916, Fujimoto, Watanabe, 1969, and Mattar, 1981) best explained LBM. The explanatory power for males was R^2^ = 83 to 84%, and that for females was R^2^ = 69%. The standard error of estimation (SEE) of the three equations showed acceptable values in both sexes. These values ranged from 0.049 to 0.080 in males and from 0.035 to 0.088 in females. The Bland–Altman concordance analysis showed adequate limits of agreement. In men, they ranged from −0.092 to 0.069 m^2^. In females, they ranged from −0.064 to 0.084 m^2^. Reference values for BSA and LBM were constructed using percentiles. **Conclusions:** This study demonstrated the validity of three equations for estimating LBM in a Chilean sample aged between 4 and 85 years. These results show consistent behavior and acceptable accuracy, especially in the Mattar equation for all ages. However, the Dubois & Dubois and Fujimoto equations could also be an alternative in females. Reference values were generated for BSA and LBM according to age and sex. The results suggest their applicability and usefulness in clinical and public health contexts.

## 1. Introduction

Fat mass (FM) and lean body mass (LBM) are fundamental estimates of body composition (BC), commonly reported in research studies, as well as in clinical and epidemiological contexts [1]. Both play a key role in contemporary body composition research in various disciplines [1,2].

LBM refers to the amount of non-fat body tissue, which includes muscle, bone, water and organs [3]. It is used as an indicator of nutritional and health status in various ages and populations [4].

Several methods have been reported in the literature to assess BC, such as dual-energy X-ray absorptiometry (DXA), air displacement plethysmography, skinfold thickness, and bioimpedance [5]. Indeed, DXA is a non-invasive and validated tool that measures BC phenotypes in a tricompartmental model (bone mass, FM and LBM) quickly and accurately [6]. Therefore, its use in research is considered one of the most widely used reference techniques for estimating the three body compartments [7].

In sum, LBM is important for posture, body movement, and physical fitness in children and adolescents [8,9] since it represents the most metabolically active mass [10]. Furthermore, it influences physical performance, motor development and overall health, especially during critical stages of growth and maturation [11]. Meanwhile, in adulthood and old age, the preservation of LBM becomes even more important, since it is closely associated with the prevention of chronic diseases, the maintenance of physical functionality and autonomy, as well as with reducing the risk of sarcopenia and improving quality of life during aging [12,13,14,15].

In recent years, thousands of studies have focused on validating anthropometric equations to predict LBM in various populations and age groups [16,17,18]. However, to our knowledge no study has explored the utility of body surface area (BSA) across all life stages, except for some studies that have suggested that BSA could be appropriate indicator to estimate LBM in youth and adults [19,20,21,22].

ASC reflects human dimensionality, predicts metabolic activity in clinical applications, and is related to metabolic heat dissipation in physiology, since heat exchange with the environment occurs mainly through the body surface [23]. Therefore, it could provide a more accurate representation of body composition, and specifically LBM, since it considers both height and weight with allometric correction, where weight is approximately proportional to a subject’s height. Therefore, the product of weight (a) × height (b) can be manipulated by a suitable constant to obtain a stable value that approximates weight [24]. This feature allows a more comprehensive assessment of metabolic mass than body weight, as it is less affected by abnormal adipose mass [25].

Consequently, based on previous evidence, this study assumes that the BSA could be an excellent predictor of LBM in a regional sample of Chile ranging from childhood, adolescence and youth to adulthood and senescence. In this regard, having a simple, non-invasive, and easy-to-apply tool such as BSA could be very useful for estimating LBM. To this end, this study provides local reference curves, adjusted to the ethnic and sociocultural reality of the Chilean population. This could contribute to the assessment and monitoring of the growth and nutritional status of children and adolescents, while in middle-aged and older adults it could help prevent and detect changes in LBM and identify sarcopenia, frailty, and risk of functional disability.

Therefore, the objectives of the study were (a) to verify the validity of a set of equations based on BSA to estimate LBM, using DXA as a reference method, and (b) to propose reference values of BSA by anthropometry and LBM by DXA according to age and sex in a regional sample of Chile from 4 to 85 years of age.

## 2. Materials and Methods

### 2.1. Type of Study and Sample

A descriptive cross-sectional study was conducted on subjects aged 4 to 85 years of both sexes in the Maule region of Chile. The sample size corresponded to 10.7% of the total population (28,840 males and 22,660 females), including 5493 participants [2868 males (5.6%) and 2625 females (5.1%)]. This ensures adequate representativeness with a 95% confidence interval and a margin of error of ±1.25%

The Human Development Index (HDI) for the Maule region in the year 2018 was 0.872. Meanwhile, for Chile it was 0.847 [26]. This indicates a high level of development on both scales, although socioeconomic gaps persist between regions at the level of Chile and neighboring countries.

Participants were recruited at different stages of the life course in different institutions. They included children aged 4 and 5 years from public preschools; schoolchildren aged 6 to 17 years enrolled in public schools; adults aged 18 to 30 years from public and private universities; adults aged 30 to 64 years from public and private institutions; and older adults (65 years and older) linked to social programs offered by the Municipality of Talca, Maule Region, Chile.

All adults who authorized and signed the informed consent form were included in the study. In the case of minors, their parents or guardians authorized their participation. Participants who had any type of physical injury that prevented walking and those who had any type of metallic implant (e.g., prosthesis, plates, screws, pacemakers, among others) were excluded because these could interfere with the accuracy of the measurements made by the DXA scanner. The study was developed in accordance with the Declaration of Helsinki for humans and was approved by the University Ethics Committee under registration UA-238.

### 2.2. Techniques and Procedures

#### 2.2.1. Anthropometry

The evaluations of the study were carried out during the years 2014 to 2018 in a laboratory of a university in the Maule region. Weight and height were initially measured, and participants were asked to wear as little clothing as possible, without shoes, wearing shorts and a t-shirt. Measurements were performed according to the recommendations of Ross, Marfell-Jones [27]. Body weight (kg) was assessed using a scale (SECA, Hamburg, Germany) with an accuracy of 0.1 kg. Standing height was measured with a stadiometer (SECA, Hamburg, Germany) with an accuracy of 0.1 cm. Evaluations of these measurements were performed twice to determine the intra-evaluator technical error of measurement (TEM). Ten percent of the total sample was evaluated, corresponding to 550 subjects of both sexes. The TEM ranged from 0.7 to 1.1%. Reliability was assessed by performing repeated measurements on the same day (re-test). Participants were invited to take the test after every 10 participants to ensure the stability of the measurements.

Seven anthropometric equations were used to estimate BSA in both sexes [28,29,30,31,32,33,34]. Table 1 shows the reference data for each equation.

#### 2.2.2. iDXA Analysis

A brand-name DXA analyzer was used (Lunar Prodigy; GE Healthcare, Madison, WI, USA) to determine fat-free mass (FFM). This equipment presents a limited scanning space for body weight of 160 kg and for height of approximately 197.5 × 66 cm. The equipment was previously calibrated every day before scanning according to the manufacturer’s instructions. Before undertaking this study, we developed and tested a pilot protocol for performing full-body scans as suggested by Nana et al. [35]. This emphasizes consistency in the positioning of subjects in the scanning area of the DXA instrument. The evaluation procedure consisted of performing a full-body scan by DXA. Previously it was verified that each participant did not have rings, metallic implants and/or pacemakers. The person being evaluated had to remain in a supine position on the densitometer table. The arms were placed extended along the body, palms down (pronation), and the legs extended in a neutral position. To ensure standard positioning and minimize movement during scanning, the ankles were secured with a Velcro strap. The equipment was operated by an evaluator with extensive DXA experience. During the study period, the same assessment team (anthropometry and DXA) was maintained. It should be noted that there were no changes in technical staff, which allowed for consistency in the assessments.

#### 2.2.3. Statistics

The normal distribution of the data used in this study was verified by means of the Kolmogorov–Smirnov test. The data were processed and calculated in Excel, SPSS 16.0 and Medcalc v.23.4. Descriptive statistics were calculated using the arithmetic mean, standard deviation and range. Differences between the two genders were verified by means of the t-test for independent samples. The relationships between the reference method (iDEXA) and the values estimated using body surface area (BSA) equations were evaluated using Pearson’s correlation coefficient (r), the coefficient of determination (r^2^), the percentage standard error of estimation (%SE), and, primarily, the standard error of estimation (SEE). The latter was considered the most appropriate indicator for comparing the validity and accuracy of estimates between different models, as it directly reflects the magnitude of the prediction error. In this regard, the SEE was used as the primary measure for comparing model performance. Meanwhile, r and r^2^ were used complementarily to describe the strength and proportion of variance explained by the observed relationships. The Bland–Altman method was used to verify the agreement between the BSA equations. The objective was the graphical representation of individual differences versus their averages, and to identify possible systematic biases or limits of agreement between the BSA equations. The concordance correlation coefficient (CCC) proposed by Lin [36] was also calculated to verify precision and accuracy.

LMS ChartMaker software version 2.316 [37] was used to develop the reference values.

The percentile curves were smoothed to create three age- and sex-specific curves according to Cole et al. [38], for example, L (lambda; skewness), M (mu; median), and S (sigma; coefficient of variation). Percentiles were calculated (P3, P5, P10, P15, P25, P50, P75, P85, P90, P95 and P97). A significance level of 0.05 was adopted in all cases.

## 3. Results

The anthropometric characteristics of weight, height, BSA and LBM values by age range and sex are shown in Table 2. There were no significant differences in body weight in the first three age groups (4 to 7 years, 8 to 11 years and 12 to 15 years). Significant differences in height were only observed in the first two groups (4 to 7 and 8 to 11 years). Significant differences were observed in older age groups. Males presented greater weight and height in relation to their female counterparts (*p* < 0.05). In relation to comparisons between BSA equations for both sexes, the same pattern was observed in all seven equations.

For example, there are no significant differences in specific ages according to sex in all BSA equations (from 4 to 7 years, from 8 to 11 years, and from 12 to 15 years). Differences appear from 16 to 19 years of age, until >80 years of age. Males presented higher BSA compared to females (*p* < 0.05).

Differences in LBM between sexes were observed from the 12–15 age group, through adolescence and adulthood, up to the >80 age group. Males had higher LBM than females (*p* < 0.05). There were no differences in LBM in the first two age groups (4 to 7 years and 8 to 11 years) (*p* > 0.05). In males and females, anthropometric values, BSA, and LBM increased from the 4 to 7 age group to around 40 to 59 years of age. They then decrease slightly at more advanced ages.

The results of the correlation analysis between the BSA equations and lean body mass (LBM) estimated using DXA (Table 3) showed high criterion validity for each sex. Among the equations analyzed, those proposed by Dubois and Dubois [28], Fujimoto et al. [31], and Mattar [34] showed the best predictive performance across all stages of life. In males, these equations had an explanatory power (r^2^) of 83–84%, while in females they reached an r^2^ of approximately 69%. However, when considering the accuracy of the estimates through the standard error of estimation (SEE), consistent and acceptable values were observed in all age groups and in both sexes. In males, the SEE ranged from 0.055 to 0.049, and in females from 0.038 to 0.088, indicating good model stability and adequate predictive capacity of LBM throughout the life cycle. When classifying the data into five age groups (4–11, 12–19, 20–39, 40–59, and ≥60 years), the three equations maintained a similar trend for each sex, although with a slight decrease in accuracy and an increase in SEE in older females, suggesting a lower predictive capacity in this group.

It should also be noted that the percentage of standard estimation error (%EE) of the three equations showed accurate values in both sexes. These values range from 2.79 to 4.57 in males and from 2.39 to 5.50 in females.

The limits of agreement between the three BSA equations are seen in the Bland–Altman plot (Dubois & Dubois [28], Fujimoto et al. [31], Mattar [34]). In males, the mean differences in BSA between the three equations ranged from −0.025 to 0.039 m^2^, with limits of agreement (lower and upper) ranging from −0.092 to 0.069 m^2^. In females, mean differences ranged from −0.001 to 0.033 m^2^, with limits of agreement ranging from −0.064 to 0.084 m^2^. In each sex, the levels of agreement between the equations were considered acceptable. In addition, there were no significant differences between the means of the three equations.

**Figure 1:**
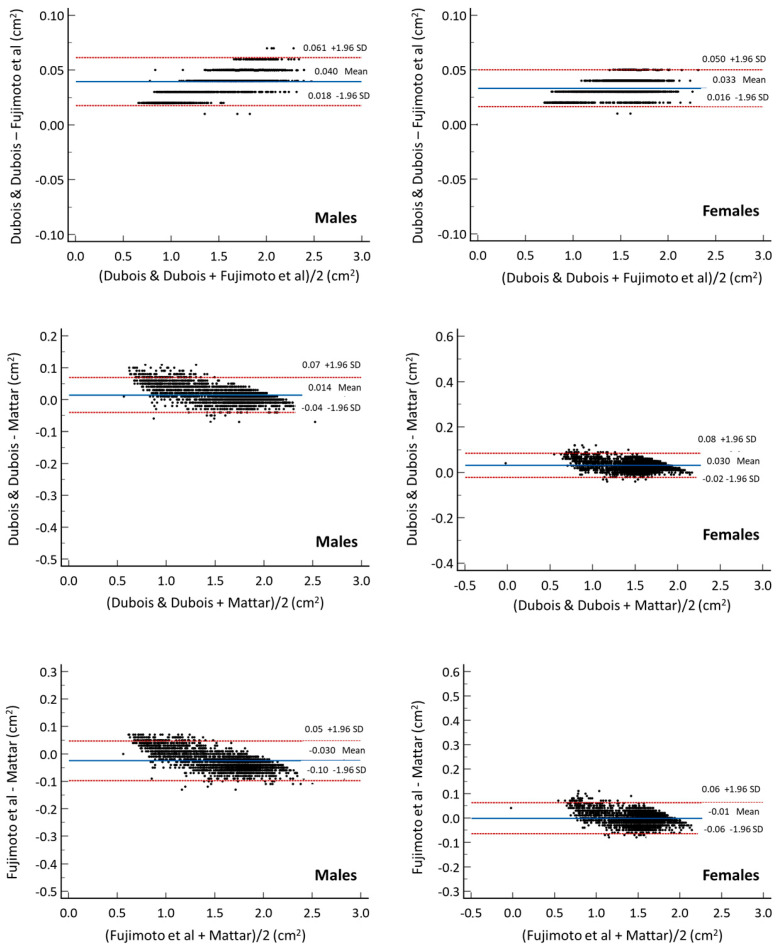
Concordance analysis (Bland–Altman plots) by sex and among the three best equations [28,31,34] for estimating BSA.

Additionally, Lin’s concordance index [36] was calculated to evaluate the precision and accuracy between the BSA equations. In both sexes, precision of 0.99 and accuracy of 0.99 were observed between the three equations (Dubois & Dubois–Fujimoto et al.; Dubois & Dubois–Mattar; Fujimoto et al.–Mattar). This demonstrates the high degree of agreement between the three equations analyzed (Figure 1).

**Figure 2:**
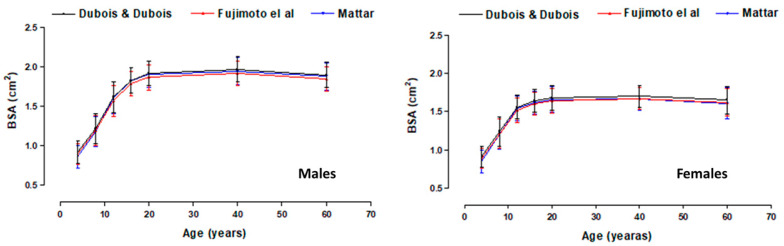
Comparison of mean BSA values of three equations [28,31,34] according to age and sex.

Figure 2 shows the comparisons of the mean values of the three BSA equations according to age and sex. For each sex, there were no significant differences between the three equations (*p* < 0.05). The three BSA equations showed similar values from 4 to 85 years of age.

Table 4 shows the BSA reference values (Mattar’s BSA equation) and Table 5 shows the LBM values determined by iDXA. In both cases they are expressed in percentiles (P3, P5, P10, P15, P25, P50, P75, P85, P90, P95 and P97), whose ages range from 4 to 85 years.

## 4. Discussion

The findings of the study have shown that the SEEs of the three equations [28,31,34] have demonstrated acceptable accuracy values. It was observed that the SEE increased progressively with age. This indicates slightly lower accuracy in both sexes at older ages. However, when the standard error of estimation (%SE) was determined in all age groups, the results reflected low relative error values (<5%). This demonstrates high accuracy in the three equations analyzed. Furthermore, the explanatory power in men was 83 to 84% and in women 69%, higher than in the other equations [29,30,32,33].

In relation to the accuracy indicator in the three BSA equations, it was observed that the SEE increased progressively with age. This indicates slightly lower accuracy in both sexes at older ages. However, when the percentage of standard estimation error (%EE) was determined in all age groups, the results reflected low relative error values. This demonstrates high accuracy in the three equations analyzed.

The explanatory power observed at each stage of life suggests that the three BSA equations can be used equivalently across different age ranges and in both sexes. However, based on the SEE accuracy of the equations that estimate BSA, for males, Mattar’s equation presented the best balance between fit (r^2^) and accuracy (SEE). Therefore, it could be considered the most generally applicable for all ages. Meanwhile, in females, lower SEE values were presented, so any of the three equations could be used. However, caution is recommended in older women due to the lower explanatory power observed.

It was also verified that there were no significant differences between the three BSA equations in both sexes and in all age ranges [28,34,39]. Furthermore, high agreement was observed among them, which supports their consistency and applicability for estimating BSA throughout the life cycle.

These findings show that the three BSA equations are interchangeable for estimating body surface area in both sexes, as they offer very high levels of precision and accuracy.

These results suggest that these equations could facilitate the assessment of LBM in clinical, sport and/or community settings where access to advanced technologies such as DXA is limited or non-existent.

BSA appears to be a good general indicator of functional body size, as its calculation is based on weight and height. This allows a positive relationship to be established with the amount of metabolically active tissue, which supports the explanatory power of LBM. Furthermore, it is a critical indicator in many medical and surgical applications, including the calculation of drug doses and the estimation of organ size at various stages of life [40].

Overall, the consistency of the explanatory power of BSA found in this study from childhood to old age suggests that this parameter can be used as a useful, rapid, inexpensive, and non-invasive tool for estimating LBM throughout the life cycle. During the growth and maturation stage, bone and muscle mass accumulation become particularly important, reaching their peak in early adulthood [41] and experiencing a progressive decrease in later stages, such as middle adulthood and senescence.

Therefore, maintaining adequate levels of LBM is essential in older age, as this contributes to preserving functionality, preventing chronic diseases and reducing the risk of functional disability associated with sarcopenia [14].

It is widely known that skeletal muscle function is critical for locomotion, bone health, neuromuscular function and metabolism. In addition, it acts as an important protein reserve under catabolic conditions [42]. Thus, the loss of skeletal muscle mass, considered one of the first signs of tissue aging, has a considerable impact on metabolism and body homeostasis [43]. Its monitoring from infancy to old age is essential to promote proper physical development and healthy aging.

From this perspective, and considering that BSA is a valid indicator to estimate LBM throughout childhood to senescence, the second objective of this study was to propose reference values of BSA obtained by anthropometry and LBM, determined by DXA, according to age and sex in a regional sample of Chile aged between 4 and 85 years.

The proposed percentiles constitute an essential tool to assess and categorize the state of BSA and LBM according to age and sex, allowing us to identify deviations from expected development, to establish population comparisons and to guide interventions in clinical, sport and epidemiological settings.

In fact, reference values are used as a way to help interpret the performance of an individual compared to a reference population [44]. Within the percentile distribution, low values of BSA and/or LBM are associated with reduced levels of LBM. Meanwhile, high values tend to be associated with higher LBM.

In this study, we adopted the cut-off points classically described by the US National Health and Nutrition Examination and Surveys (NHANES) [45,46] for anthropometric curves for both sexes and all ages (with <p10 as low, p10 to p85 as adequate and >p85 as elevated).

These specific values help to elucidate age-related changes associated with health problems [47]. It even allows a more precise characterization of people at higher risk of disability and morbidity so that they can receive specific support [48].

Thus, the use of cut-off points based on percentiles allows not only monitoring of nutritional status, body size and LBM throughout the life cycle. It also allows early identification of individuals who could benefit from targeted interventions. In particular, values below the 10th percentile could present a higher risk of functional decline, malnutrition, or sarcopenia.

It was not possible to record food consumption and physical activity levels. Secondly, hydration status (overhydration and/or dehydration) was not measured in the sample studied, as suggested by Nana et al. [35]. The absence of this information could influence body composition, especially LBM, and consequently the estimation of BSA. Thirdly, the cross-sectional design of the study prevents causal relationships from being established between the variables analyzed. Therefore, future longitudinal studies should be planned.

It is also necessary to highlight the strengths of the study. For example, it is the first study carried out in Chile and South America that validates BSA equations as predictors of LBM in a broad sample and age range. Second, the results obtained here can serve as a baseline for future comparisons in secular trend studies. Third, the use of DXA as a reference method represents a relevant methodological strength to validate the BSA equations as it is a highly accurate and reliable method for the assessment of body composition (LBM). Fourth, the development of percentiles represents a valuable tool in the field of health sciences. It allows estimation of LBM both through field tests, such as BSA, and through laboratory methods, such as DXA scanning. Calculations for both methods can be performed at the following link: www.reidebihu.net/asc.php (accessed on 15 November 2025).

## 5. Conclusions

This study demonstrated that, of the seven equations used to calculate BSA, three were valid for estimating LBM in the Chilean population aged between 4 and 85 years.

In particular, the Mattar equation could be an alternative for general use and application due to its greater accuracy for both sexes. However, the Dubois & Dubois and Fujimoto equations could also be an alternative for women. In addition, the study provides reference values for BSA and LBM according to age and sex. The results suggest its applicability and usefulness in clinical and epidemiological contexts. This information allows for nutritional surveillance and monitoring throughout the life cycle. This is especially true in relation to LBM, which facilitates assessment and monitoring at different stages of life.

## Figures and Tables

**Table 1:** Equations estimating BSA.

*n*°	Authors	Years	Subjects	Equation
1	Dubois & Dubois [28]	1916	All ages	BSA = 0.007184 × weight (kg)^0.425^ × height (cm)^0.725^
2	Boyd [29]	1935	Children	BSA = 4.688 × weight (kg)^(0.8168−0.0154×log weight (kg))^
3	Costeff [30]	1966	All ages	BSA = (4 × weight (kg) + 7)/90 + weight (kg)
4	Fujimoto et al. [31]	1969	All ages	BSA = 0.008883 × Weight^0.444^ × Height^0.663^
5	Haycock et al. [32]	1978	All ages	BSA = 0.024265 × Weight^0.5378^ × Height^0.3964^
6	Mosteller [33]	1987	Adults	BSA = √(weight (kg) × height (cm))/3600
7	Mattar [34]	1981	Adults	BSA = (Height + Weight − 60)/100

Legend: BSA: Body surface area.

**Table 2:** Values of anthropometric variables, lean body mass, and body surface area of the study sample.

Age (Years)	*n*	Anthropometry				BSA Equations														DXA	
		Weight (kg)		Height (cm)		Dubois & Dubois [28] (m^2^)		Boyd [29] (m^2^)		Costeff [30] (m^2^)		Fujimoto et al. [31] (m^2^)		Haycock et al. [32] (m^2^)		Mosteller [33] (m^2^)		Mattar [34] (m^2^)		LBM (kg)	
		X	SD	X	SD	X	SD	X	SD	X	SD	X	SD	X	SD	X	SD	X	SD	X	SD
Males																					
4–7	300	26.1	6.8	120.2	8.2	0.92	0.1	0.95	0.2	0.95	0.2	0.9	0.1	0.93	0.2	0.93	0.1	0.86	0.1	19.21	5.5
8–11	498	39.1	11	139.1	9.3	1.22	0.2	1.24	0.2	1.25	0.2	1.19	0.2	1.22	0.2	1.22	0.2	1.18	0.2	27.16	6.9
12–15	754	57.9	12	163.2	11	1.62	0.2	1.62	0.2	1.6	0.2	1.57	0.2	1.61	0.2	1.61	0.2	1.61	0.2	44.46	9.6
16–19	784	71.3	12	171.8	6.8	1.83	0.2	1.85	0.2	1.8	0.2	1.79	0.2	1.85	0.2	1.84	0.2	1.83	0.2	55.27	7.6
20–39	362	77.9	12	173.4	7	1.92	0.2	1.95	0.2	1.89	0.2	1.87	0.2	1.94	0.2	1.93	0.2	1.91	0.2	58.66	9.0
40–59	75	85.8	13	169.7	6.2	1.97	0.2	2.04	0.2	1.98	0.2	1.92	0.2	2.03	0.2	2.01	0.2	1.95	0.2	57.92	8
60–79	75	80.9	13	167	6.6	1.9	0.2	1.97	0.2	1.92	0.2	1.85	0.2	1.95	0.2	1.86	0.2	1.88	0.2	53.72	9.4
>80 y	20	72.2	3	163.6	4.5	1.78	0.1	1.84	0.1	1.82	0	1.74	0.1	1.83	0.1	1.71	0.2	1.76	0.1	49.12	3.7
Females																					
4–7	248	25.8	6	119.4	11	0.91	0.1	0.94	0.1	0.94	0.2	0.89	0.1	0.92	0.1	0.92	0.1	0.85	0.2	17.8	4.4
8–11	438	40.2	10	141.1	12	1.24	0.2	1.27	0.2	1.27	0.2	1.21	0.2	1.25	0.2	1.25	0.2	1.21	0.2	26.19	6.0
12–15	427	57	11	157.2 *	6.8	1.56	0.2	1.59	0.2	1.58	0.2	1.52	0.2	1.58	0.2	1.57	0.2	1.54	0.2	37.42 *	6.2
16–19	360	62.9 *	12	158.9 *	5.6	1.64 *	0.2	1.69 *	0.2	1.68 *	0.2	1.60 *	0.2	1.67 *	0.2	1.66 *	0.2	1.62 *	0.2	39.56 *	5.3
20–39	313	65.68 *	13	160.4 *	6.6	1.68 *	0.2	1.73 *	0.2	1.72 *	0.2	1.64 *	0.2	1.72 *	0.2	1.7 *	0.2	1.66 *	0.2	41.45 *	7.6
40–59	278	70.6 *	13	156.4 *	7.9	1.7 *	0.1	1.79 *	0.2	1.79 *	0.2	1.67 *	0.1	1.77 *	0.2	1.74 *	0.2	1.67 *	0.2	41.22 *	7.4
60–79	539	69.1 *	12	152.2 *	6.5	1.65 *	0.2	1.75 *	0.2	1.77 *	0.2	1.62 *	0.2	1.72 *	0.2	1.71 *	0.2	1.61 *	0.2	37.78 *	9.2
>80 y	22	66.7 *	14	153.5 *	7.3	1.64 *	0.2	1.72 *	0.2	1.73 *	0.2	1.61 *	0.2	1.7 *	0.2	1.69 *	0.2	1.6 *	0.2	35.50 *	5.2

Legend. X: mean; SD: standard deviation; BSA: body surface area; DXA: dual X-ray absorptiometry; LBM: lean body mass; *: significant differences between age groups.

**Table 3:** Criterion validity analysis of the equations estimating BSA as an indirect predictor of LBM for both genders.

BSA/Age (Years)			Males						Females			
	X	SD	R	R^2^	SEE	%EE	X	SD	R	R^2^	SEE	%EE
Dubois & Dubois [28]												
4.0–11.9 y	1.10	0.22	0.761	0.58	0.049	4.39	1.12	0.23	0.855	0.73	0.035	3.13
12.0–19.9 y	1.73	0.21	0.786	0.62	0.063	3.66	1.60	0.16	0.771	0.59	0.038	2.35
20.0–39.9 y	1.92	0.16	0.613	0.38	0.080	4.15	1.68	0.16	0.546	0.30	0.064	3.77
40.0–59.9	1.97	0.16	0.733	0.54	0.055	2.80	1.70	0.14	0.608	0.37	0.059	3.46
>60 y	1.88	0.16	0.664	0.44	0.068	3.63	1.65	0.18	0.492	0.24	0.088	5.33
All ages	1.59	0.37	0.910	0.83	0.061	3.85	1.51	0.30	0.83	0.69	0.055	3.66
Fujimoto et al. [31]												
4.0–11.9 y	1.08	0.22	0.760	0.58	0.049	4.50	1.10	0.23	0.855	0.73	0.035	3.20
12.0–19.9 y	1.68	0.21	0.784	0.61	0.063	3.77	1.56	0.16	0.769	0.59	0.038	2.41
20.0–39.9 y	1.87	0.16	0.611	0.37	0.080	4.26	1.64	0.16	0.543	0.30	0.064	3.87
40.0–59.9	1.92	0.15	0.733	0.54	0.055	2.86	1.67	0.14	0.609	0.37	0.059	3.52
> 60 y	1.84	0.16	0.663	0.44	0.069	3.72	1.62	0.18	0.493	0.24	0.088	5.43
All ages	1.55	0.36	0.911	0.83	0.061	3.95	1.47	0.29	0.833	0.69	0.055	3.75
Mattar [34]												
4.0–11.9 y	1.06	0.23	0.761	0.58	0.049	4.57	1.08	0.25	0.846	0.72	0.036	3.34
12.0–19.9 y	1.72	0.21	0.791	0.63	0.063	3.63	1.58	0.16	0.767	0.59	0.038	2.39
20.0–39.9 y	1.91	0.17	0.614	0.38	0.079	4.15	1.66	0.17	0.548	0.30	0.063	3.82
40.0–59.9	1.95	0.17	0.739	0.55	0.055	2.79	1.67	0.15	0.618	0.38	0.058	3.49
>60 y	1.87	0.17	0.668	0.45	0.068	3.65	1.61	0.20	0.484	0.24	0.088	5.50
All ages	1.57	0.39	0.922	0.84	0.060	3.82	1.47	0.31	0.833	0.69	0.055	3.76

Legend: BSA: body surface area, SEE: standard estimation error, %EE: percentage of standard estimation error.

**Table 4:** Percentile distribution of body surface area (m^2^) by age range and sex.

	L	M	S	P3	P5	P10	P15	P25	P50	P75	P85	P90	P95	P97
	Males													
4	−0.0074	0.669	0.0012	0.54	0.55	0.58	0.59	0.62	0.67	0.73	0.77	0.79	0.83	0.86
5	−0.0064	0.7585	0.0012	0.61	0.63	0.65	0.67	0.7	0.76	0.83	0.87	0.89	0.94	0.97
6	−0.0055	0.8485	0.0012	0.69	0.7	0.73	0.75	0.78	0.85	0.92	0.97	1.00	1.05	1.08
7	−0.0044	0.939	0.0012	0.76	0.78	0.81	0.83	0.87	0.94	1.02	1.07	1.1	1.15	1.19
8	−0.0033	10.308	0.0012	0.83	0.86	0.89	0.92	0.95	1.03	1.12	1.17	1.2	1.26	1.3
9	−0.0022	11.268	0.0012	0.91	0.94	0.97	1.00	1.04	1.13	1.22	1.27	1.31	1.37	1.41
10	−0.0012	12.282	0.0011	1.00	1.02	1.06	1.09	1.14	1.23	1.33	1.38	1.42	1.48	1.52
11	−0.0002	13.332	0.0011	1.08	1.11	1.16	1.19	1.24	1.33	1.44	1.5	1.54	1.6	1.64
12	0.0007	14.393	0.0011	1.17	1.2	1.25	1.29	1.34	1.44	1.55	1.61	1.65	1.72	1.76
13	0.0012	15.426	0.001	1.27	1.3	1.35	1.38	1.44	1.54	1.65	1.72	1.76	1.83	1.87
14	0.0011	16.377	0.001	1.35	1.39	1.44	1.47	1.53	1.64	1.75	1.82	1.86	1.93	1.97
15	0.0005	17.185	0.001	1.43	1.46	1.52	1.55	1.61	1.72	1.83	1.9	1.95	2.01	2.06
16	−0.0004	17.811	0.0009	1.49	1.53	1.58	1.62	1.67	1.78	1.9	1.96	2.01	2.08	2.13
17	−0.0014	18.257	0.0009	1.54	1.57	1.63	1.66	1.72	1.83	1.94	2.01	2.05	2.12	2.17
18	−0.0023	18.569	0.0009	1.58	1.61	1.66	1.7	1.75	1.86	1.97	2.04	2.08	2.15	2.2
19	−0.003	18.793	0.0009	1.6	1.63	1.68	1.72	1.77	1.88	1.99	2.06	2.11	2.18	2.22
20–39	−0.0036	18.954	0.0009	1.62	1.65	1.7	1.74	1.79	1.9	2.01	2.07	2.12	2.19	2.24
40–59	−0.004	19.055	0.0008	1.63	1.66	1.71	1.75	1.8	1.91	2.02	2.08	2.13	2.2	2.25
60–79	−0.0043	1.911	0.0008	1.64	1.67	1.72	1.75	1.81	1.91	2.02	2.09	2.13	2.2	2.25
>80	−0.0045	1.915	0.0008	1.65	1.68	1.73	1.76	1.81	1.91	2.03	2.09	2.13	2.2	2.25
Females														
4	−0.0011	0.6711	0.0012	0.53	0.55	0.57	0.59	0.62	0.67	0.73	0.76	0.79	0.82	0.85
5	0.0004	0.7633	0.0012	0.61	0.63	0.65	0.67	0.7	0.76	0.83	0.86	0.89	0.93	0.96
6	0.0018	0.8573	0.0012	0.68	0.7	0.73	0.76	0.79	0.86	0.93	0.97	1.00	1.04	1.07
7	0.0032	0.9547	0.0012	0.76	0.78	0.82	0.84	0.88	0.95	1.03	1.08	1.11	1.15	1.18
8	0.0046	1.056	0.0012	0.84	0.87	0.91	0.93	0.98	1.06	1.14	1.19	1.22	1.27	1.3
9	0.0058	11.606	0.0011	0.92	0.95	1.00	1.03	1.07	1.16	1.25	1.3	1.34	1.39	1.42
10	0.0068	12.646	0.0011	1.01	1.04	1.09	1.12	1.17	1.26	1.36	1.41	1.45	1.5	1.54
11	0.0075	13.618	0.0011	1.09	1.12	1.17	1.21	1.26	1.36	1.46	1.52	1.56	1.61	1.65
12	0.0079	14.456	0.0011	1.16	1.2	1.25	1.29	1.34	1.45	1.55	1.61	1.65	1.71	1.74
13	0.0078	15.112	0.0011	1.22	1.26	1.31	1.35	1.4	1.51	1.62	1.68	1.72	1.78	1.82
14	0.007	15.593	0.001	1.27	1.3	1.36	1.4	1.45	1.56	1.67	1.73	1.77	1.83	1.87
15	0.0054	15.928	0.001	1.3	1.34	1.39	1.43	1.49	1.59	1.7	1.76	1.8	1.87	1.91
16	0.0032	16.151	0.001	1.33	1.37	1.42	1.46	1.51	1.62	1.72	1.79	1.83	1.89	1.93
17	0.0007	16.294	0.001	1.36	1.39	1.44	1.47	1.53	1.63	1.74	1.8	1.84	1.91	1.95
18	−0.002	16.387	0.001	1.37	1.4	1.45	1.49	1.54	1.64	1.75	1.81	1.85	1.92	1.97
19	−0.0045	16.433	0.0009	1.39	1.41	1.46	1.49	1.54	1.64	1.75	1.82	1.86	1.93	1.98
20–39	−0.0067	16.412	0.0009	1.39	1.42	1.46	1.49	1.54	1.64	1.75	1.81	1.86	1.93	1.98
40–59	−0.0086	16.308	0.0009	1.39	1.41	1.46	1.49	1.53	1.63	1.74	1.8	1.85	1.92	1.97
60–79	−0.0103	16.134	0.0009	1.37	1.4	1.44	1.47	1.52	1.61	1.72	1.79	1.83	1.91	1.96
>80	−0.012	15.944	0.0009	1.36	1.39	1.43	1.46	1.5	1.59	1.7	1.77	1.81	1.89	1.94

Legend: L: asymmetry (lambda), M: median, and S: coefficient of variation (sigma).

**Table 5:** Percentile distribution of lean mass (kg) by age range and sex.

Age	L	M	S	P3	P5	P10	P15	P25	P50	P75	P85	P90	P95	P97
	Males													
4	−1.789	14.09	0.1999	10.57	10.88	11.41	11.81	12.49	14.09	16.44	18.25	19.85	23.14	26.3
5	−14.052	159.255	0.1987	11.79	12.17	12.81	13.29	14.09	15.93	18.47	20.31	21.83	24.67	27.05
6	−10.211	177.979	0.1975	12.99	13.44	14.21	14.78	15.71	17.8	20.54	22.39	23.85	26.41	28.39
7	−0.6372	197.242	0.1961	14.16	14.71	15.62	16.29	17.37	19.72	22.65	24.52	25.94	28.3	30.03
8	−0.2552	218.182	0.1944	15.38	16.04	17.14	17.93	19.18	21.82	24.93	26.83	28.22	30.46	32.03
9	0.1264	242.611	0.1922	16.76	17.57	18.89	19.83	21.29	24.26	27.59	29.53	30.92	33.08	34.55
10	0.5009	272.184	0.1891	18.4	19.41	21.02	22.14	23.86	27.22	30.8	32.81	34.21	36.34	37.76
11	0.8521	307.896	0.1849	20.38	21.65	23.63	24.98	26.99	30.79	34.66	36.77	38.21	40.35	41.76
12	11.715	348.865	0.1796	22.69	24.28	26.68	28.28	30.61	34.89	39.07	41.28	42.77	44.96	46.37
13	14.455	392.445	0.1732	25.27	27.19	30.03	31.88	34.53	39.24	43.72	46.03	47.57	49.82	51.25
14	16.537	434.999	0.1659	28.05	30.26	33.47	35.53	38.44	43.5	48.2	50.61	52.19	54.49	55.94
15	17.955	472.828	0.1584	30.87	33.27	36.73	38.92	41.99	47.28	52.14	54.6	56.22	58.55	60.03
16	1.878	503.156	0.1514	33.48	35.97	39.53	41.78	44.92	50.32	55.24	57.73	59.37	61.72	63.21
17	19.067	525.393	0.1453	35.71	38.19	41.74	43.99	47.14	52.54	57.48	59.98	61.61	63.97	65.46
18	18.885	541.051	0.1403	37.55	39.96	43.43	45.65	48.75	54.11	59.03	61.52	63.16	65.53	67.02
19	18.371	551.457	0.1364	38.98	41.29	44.66	46.82	49.86	55.15	60.04	62.53	64.17	66.54	68.05
20–39	17.662	557.421	0.1333	40.03	42.25	45.49	47.58	50.54	55.74	60.59	63.08	64.72	67.09	68.6
40–59	16.892	559.433	0.1304	40.76	42.86	45.97	47.99	50.86	55.94	60.72	63.19	64.82	67.18	68.69
60–79	1.613	559.143	0.1277	41.27	43.27	46.24	48.18	50.96	55.91	60.61	63.04	64.65	67	68.49
>80	15.371	558.224	0.125	41.7	43.6	46.45	48.32	51	55.82	60.43	62.82	64.41	66.74	68.22
Females														
4	−14.802	124.277	0.1924	9.3	9.59	10.07	10.44	11.04	12.43	14.35	15.74	16.89	19.04	20.87
5	−11.996	143.388	0.1902	10.65	10.99	11.58	12.01	12.73	14.34	16.48	17.96	19.13	21.22	22.88
6	−0.9176	16.301	0.188	12	12.42	13.11	13.63	14.46	16.3	18.65	20.2	21.4	23.45	24.99
7	−0.6357	183.935	0.1857	13.42	13.91	14.74	15.34	16.31	18.39	20.96	22.58	23.81	25.83	27.3
8	−0.359	207.033	0.1831	14.96	15.55	16.53	17.23	18.35	20.7	23.49	25.2	26.45	28.47	29.9
9	−0.0977	233.007	0.1801	16.7	17.4	18.55	19.37	20.65	23.3	26.33	28.13	29.43	31.47	32.88
10	0.1339	261.335	0.1767	18.6	19.43	20.76	21.71	23.17	26.13	29.41	31.32	32.67	34.76	36.18
11	0.3191	290.277	0.173	20.59	21.54	23.07	24.13	25.77	29.03	32.55	34.56	35.96	38.11	39.56
12	0.4497	316.974	0.1689	22.5	23.56	25.24	26.41	28.2	31.7	35.42	37.52	38.97	41.18	42.66
13	0.5244	338.785	0.1646	24.16	25.3	27.09	28.33	30.22	33.88	37.74	39.89	41.38	43.64	45.14
14	0.5493	355.188	0.1602	25.55	26.72	28.56	29.84	31.77	35.52	39.45	41.64	43.15	45.43	46.94
15	0.5312	366.847	0.1559	26.67	27.85	29.7	30.98	32.92	36.68	40.64	42.83	44.35	46.65	48.17
16	0.4836	374.865	0.1517	27.57	28.73	30.56	31.83	33.75	37.49	41.42	43.62	45.14	47.45	48.98
17	0.424	380.398	0.1481	28.28	29.42	31.21	32.46	34.35	38.04	41.95	44.14	45.66	47.96	49.5
18	0.3699	384.462	0.1452	28.83	29.94	31.71	32.93	34.8	38.45	42.33	44.51	46.02	48.33	49.87
19	0.3358	387.136	0.143	29.2	30.3	32.04	33.25	35.1	38.71	42.57	44.74	46.25	48.55	50.09
20–39	0.3334	387.587	0.1417	29.32	30.41	32.14	33.34	35.17	38.76	42.58	44.73	46.23	48.51	50.03
40–59	0.3689	384.961	0.1411	29.12	30.2	31.93	33.12	34.94	38.5	42.27	44.39	45.86	48.1	49.58
60–79	0.4274	379.892	0.1408	28.68	29.77	31.48	32.67	34.48	37.99	41.7	43.77	45.2	47.38	48.83
>80	0.4891	374.306	0.1407	28.19	29.28	30.99	32.18	33.96	37.43	41.07	43.09	44.49	46.6	48.01

Legend: L: asymmetry (lambda), M: median, and S: coefficient of variation (sigma).

## Data Availability

Data supporting the findings of this study are available by contacting the corresponding author.
